# 

**Published:** 1895-01

**Authors:** 


### INDEX

TO THE

### HOMŒOPATHIC PHYSICIAN.

### VOLUME XV, 1895.

PAGE
Abbreviations of Remedies used, Table of, in the Repertory of Scarlet Fever .... 72
Abstinence, Remarkable Case of .... 562
Accoucheur's Emergency Manual. By W. A. Yingling, M. D. Review of 196
Acids of Fruits, The. G. W. Johnson.. 242
Acute Cases, Two. C. L. Olds, M. D... 98
Aggravation from Jarring the Bed. Walter M. James, M. D .... 57
Aggravation from Jarring. E. V. Ross, M. D .... 179
Alexander III, Autopsy of .... 85
Allen, J. H., M. D. Artificial Diseases and Their Treatment .... 219
Divinity of the Law .... 26
A Protest from the I. H. A .... 36
Syphilinum or Luesinum, with comments .... 353
"The Truth Shall make us Free" 133, 263
Alumni Association of the Hahnemann Medical College, Philadelphia, The. Note .... 198
Amalgam Fillings. A consideration of some of the objections by physicians to. Charles H. Taft, M. D .... 120
American Association of Orificial Surgeons .... 391
American Institute of Homœopathy, The .... 144, 199, 238, 392
American Institute of Homœopathy. A. K. Crawford, M. D .... 238
American Institute of Homœpathy, Transactions of: Review of .... 573
American Medical Publishers' Association, The .... 200, 296
Annales d'Oculistique. By Drs. E. Valude and D. E. Sultzer. Review of .... 197
Antisepsis and Antiseptics. By Charles Milton Buchanan. Review of... 435
Anti-Vaccination. Montague R. Leverson, M. D. .... 433
Aortic Insufficiency. Lawrence M. Stanton, M. D .... 73
Apis-Mellifica. Walter M. James, M. D .... 105, 153, 201
Apology, An. Walter M. James, M. D. 249
Apology Number Two. Walter M. James, M. D .... 297

PAGE
Appendicitis and the Grape-Seed .... 295
Appendicitis, Repertory on. W. A. Yingling, M. D .... 273
Archives of Pediatrics, The. Review of .... 583
Arsenicum-Album. Walter M. James, M. D .... 345, 393, 441, 489, 537
Art Amateur .... 52
Artificial Diseases and Their Treatments. J. H. Allen, M. D .... 219
Aurum in Sarcocele .... 382
Autopsy of Alexander III, Emperor of Russia. Translated by Frederic Preston, M. D .... 85

Bacon, Sir Francis. Cipher Story. By Orville W. Owen, M. D .... 246, 342
Banerjee, Dr. D. N. Vaccination and Small-pox .... 321
Baylies, B. L. B., M. D. A Protest from the I. H. A .... 36
Bellis-perennis. Accidental Proving of. Edmund Carleton, M. D .... 473
Bellis-perennis. Wm. G. Dietz, M. D... 500
Billings, Robert A., M. D. Symptoms from Rhus Poisonings .... 369
Bœricke, Dr. F. E .... 440
Boger, C. M., M. D., Repertory of Symptoms of Ovaries .... 540
Therapeutic Hints .... 194, 241, 383
Book Notices...48, 102, 146, 196, 242, 289, 294, 342, 383, 435, 448, 527, 570
Boy's Essay on Breath, A .... 56
Brady, Edward F., M. D. The Committee on Legislation and the Promotion of Homœopathy .... 99
Bronchitis: Capillary, with Brain Symptoms Cured with Stramonium .... 447
Bureau of *The Organon* and Homœopathic Philosophy, A .... 294
Butler, Clarence Willard, M. D. The Society of Homœopathicians .... 41

Carleton, Edmund, M. D. Accidental Provings of Bellis-perennis .... 473
Interstitial Fibroid of Uterus .... 297
A Plausible Fallacy .... 401
Prominent Remedies in the Recent Epidemic of Influenza .... 170
Carr, A. P., M. D. In Memoriam, Rev. C. P. Jennings, S. T. D., M. D .... 45

PAGE

Cases Cured by Alexander Villers, M. D. Translated by A. McNeil, M. D. 182
Cases Cured by Dr. Hesse. A. M. McNeil, M. D. 515
Cases from Practice. Lawrence M. Stanton, M. D. 73
Cases, Two Acute. C. L. Olds, M. D. 98
Chap-Book, The. Review of 102
Children's Homœopathic Hospital of Philadelphia 198, 295
Clark, G. E., M. D. What is a Homœopathic Prescription? 417
Clark, W. B., M. D. Dr. Constantine Hering on Vaccination 232
Climate and Health. Review of...82, 285, 533, 573
Clinics at Cook County Hospital 344
Clinical Case. F. S. Davis, M. D. 473
Clinical Cases. Olin M. Drake, M. D. 475
Clinical Cases. J. R. Haynes, M. D. 455
Clinical Cases. A. L. Kennedy, M. D. 444
Clinical Cases. F. O. Pease, M. D. 458
Clinical Notes and Reflections. Jos. Fitz Mathew, M. D. 79
Close, Stuart, M. D. Imagination in Medicine 479
Colburn, Dr. L. E. 151
Communion, The Individual Cup at. John L. Moffat, M. D. 145
Convulsions: Hypericum for. A. S. Ironside 568
Cook County Hospital 151, 344
Cornu-cutaneum. Lawrence M. Stanton, M. D. 75
Corrections 535
Coryza. Lawrence M. Stanton, M. D. 74
Cranch, Edward, M. D. First Impressions of a Medical Examiner 173
Lithæmia and Psora 507
Crawford, A. K., M. D. American Institute of Homœopathy 238
Cundurango, Verification of. Dr. W. Goullon 249
Cyclamen 194

Dake, Dr., the Eulogium of. G. J. Waggoner, M. D. 145
The Eulogium of. S. F. Shannon, M. D. 193
Davis, F. S., M. D., Clinical Case 473
Principles 397
Defective Speech and Deafness, by Lillie Eginton Warren. Review of. 48
Dermatology, Quarterly Atlas of. 533
Dever, I., M. D. In Memoriam. Dr. Joshua Emmons 47
Dictionary of Medicine, New Pronouncing. By John M. Keating, M. D. Review of 293
Dictionary, Medical, Gould's 150
Dictionary, The Standard, of Funk & Wagnalls 52, 242, 344, 390, 534
Dietz, Wm. G., M. D., Bellus-perennis 500
Dillingham, Dr., has removed 535
Diphtheria, a Case of. S. Mills Fowler, M. D. 472
Diseases of the Heart and Arteries; Their Causes, Nature and Treatment. By John H. Clark, M. D. Review of 529
Diseases of the Nose and Throat. By Watson Williams, M. D. Notice of 53
Diseases of Women. By Henry J. Garrigues, M. D. Review of 294

PAGE

Divinity of the Law. J. H. Allen, M. D. 26
Drake, Olin M., M. D. Clinical Cases 475
Experiences in the Treatment of Gonorrhœa 157
The First Prescription 3
Dunham Medical College 575

Editorials, 1, 57, 105, 153, 201, 249, 297, 345, 393, 441, 489, 537
Editorial, Vaccination. Walter M. James, M. D. 1
Effects, Primary, Indifferent, and Secondary. A. McNeil, M. D. 299
Electro-therapeutists, National Society of 343
Engelbach, Dr. Theodore 151
Essentials of Homœopathic Therapeutics. By W. A. Dewey, M. D. 572
Essex, Earl of; Tragical History of. Review of 383
Everly, W. E., M. D. Labor Pains Treated with the Simillimum 233
Evolution in Medicine; The Use of Association. W. H. Leonard, M. D. 39
Eye, Diseases of: Hand-book of. By Dr. A. Eugene Fink 390

Fibroid of Uterus, Interstitial. Edmund Carleton, M. D. 297
Fincke, B., M. D. Paracelsus and Hahnemann 491
Points on the repeal of the Compulsory Vaccination Law of the State of New York 228
The Society of Homœopathicians—A Protest 39
First Impressions of a Medical Examiner. Edward Cranch, M. D. 173
First Prescription, The. Olin M. Drake, M. D. 3
Fitz Mathew, Jos., M. D. Clinical Notes and Reflections 79
Pure Homœopathy *vs.* Electicism. 43
Flatulent Dyspepsia. Lawrence M. Stanton, M. D. 75
For Sale 536
Fowler, S. Mills, M. D. A case of Diphtheria 472
Mitral Incompetency—With Consequent Pulmonary Lesion 467
Fruits: Acids of 242
Fun for Doctors 56
Funk & Wagnalls' Standard Dictionary 52, 242, 344, 390, 534

Gatchell, Dr. Charles 248
Genito-Urinary and Venereal Diseases, Carlton's 566
Gentry, Dr. Wm. D 200, 391
Gentry's Record of the Homœopathic Materia Medica. By William Gentry, M. D. 104
Gonorrhœa, Experiences in the Treatment of. Olin M. Drake, M. D. 157
Gould's New Medical Dictionary. Review of 150
Goullon, Dr. U. Silicea in Affections of the Eustacian Tube 288
Verification of Cundrango 249
Gout and Its Cure By J. Compton Burnett, M. D. 389
Graphites 194

Hahnemann Medical College of Philadelphia. Alumni Association 198

PAGE
Hahnemann, Portrait of.................... 296
Hahnemann's Therapeutics. Hints by R. E. Dudgeon, M. D. Review of. 531
Hand-Book of Diseases of the Eye. By Dr. A. Eugen Fink. Notice of... 390
Harrington, Mark W. Information Relative to the Investigation of the Influence of Climate on Health................................ 285
Sanitary Climatology.................. 82
Haynes, J. R., M. D. Clinical Cases... 455
The Mind Symptoms of Our Homœopathic Drugs..................... 450
What are we to understand by the Term Homœopathy?.................. 319
Headache Cured by Nat-mur. J. T. O'Connor, M. D...................... 567
Heath. Alfred. M. D Urita Moroides, or Laportea Moroides.................. 100
Hering, Dr. Constantine, on Vaccination.............................. 232
Hering College............................ 536
Heroic Homœopathy......................... 56
Hesse, Cases Cured by Dr................ 515
High Potencies, The Value of. Theo. H. Winans, M. D. ..................... 77
History of Medicine, The. By Roswell Park, M. D............................. 150
Holcombe, A. W., M. D. Spasms and Convulsions.........................322, 378
Homœopathic Annual for 1894. Review of.................................. 527
Homœopathic Eye, Ear, and Throat Journal, The. Review of............ 148
Homœopathic Materia Medica on a New and Original Plan, A. By M. W. Van Denburg, M. D. Review of..................................... 292
Homœopathic Medical College of Michigan................................. 536
Homœopathic Medical College of Missouri.................................. 192
Homœopathic Medical Society of New York.................................. 440
Homœopathic Medical Society of Chicago, The................................ 152
Homœopathic Medical Society of Ohio, The.................................... 152
Homœopathic Medical Society of Pennsylvania. The...................... 343
Homœopathic Prescription? What is a. G. E. Clark, M. D............... 417
Homœopathic Philosophy, A Bureau of........................................ 294
Homœopathicians, Society of.....39, 41, 141, 298, 300, 397
Homœopathicians, The Society of. Clarence Willard Butler, M. D.... 41
Homœopathicians, The Society of—A Protest. B. Fincke, M. D.......... 89
Homœopathicians, Society of. A McNeil, M. D................................ 298
Homœopathy. By John H. Clarke, M. D. Review of....................... 148
Homœopathy, American Institute of. A. K. Crawford, M. D................ 238
Homœopathy, The Champion of. O. T. Huebener, M. D..................... 195
Homœopathy, The Committee on Legislation and the Promotion. Edward F. Brady, M. D.................. 99
Homœopathy and Correlated Subjects, Thoughts on the Philosophy of. J. W. Thompson, M. D............... 301
Homœopathy Failed, Where. A. McMcNeil, M. D................................ 494

PAGE
Homœopathy in Missouri Wm. C. Richardson, M. D......................... 234
Homœopathy, Pure, vs. Eclecticism. Jos. Fitz Mathew, M. D.............. 43
Homœopathy. What are We to Understand by the Term? J. R. Haynes, M. D.................................. 319
Hoopes, L., M. D. Vaccination........... 267
Hospital, Children's Homœopathic. 198, 295
Housemaid's Knee. Lawrence M. Stanton, M. D.................................. 76
Huebener, O. T., M D. The Champion of Homœopathy.......................... 195
Hydrogen Peroxide. Medicinal. The Real Value of................................ 53
Hydrogen Peroxide: Therapeutical Applications of. Charles Marchand........................................ 247
Hypericum for Convulsions. A. S. Ironside.......................................... 568

Ide, Dr. Henri G................................ 535
Ideal Sanitation of a Physician's Office. B. W. James, M. D....................... 548
Imagination in Medicine. Stuart Close, M. D................................................ 479
Index Medicus. A Monthly Journal by Drs. John T. Billings and Robert Fletcher. Review of...... 50
Influenza. Prominent Remedies in the Recent Epidemic of. Edmund Carleton, M. D.................. 170
Information Relative to the Investigation of the Influence of Climate on Health. Mark W. Harrington. 288
In Memoriam. Dr. Joshua Emmons. I. Dever, M. D............................ 47
Rev. C. P. Jennings, S. T. D., M. D. A. B. Carr, M D......................... 45
Dr. Guy A. T. Lincoln. A. L. K. 506, 565
Dr. Mahlon Preston. Walter M. James, M. D.................................. 501
Dr. Mahlon Preston. W. A. D. Pierce, M. D.................................. 505
International Hahnemannian Association—Address to members. A. R. Morgan, M. D................................ 83
International Hahnemannian Association, a Protest from the.............. 36
International Hahnemannian Association, The..................................... 343
International Medical Annual and Practitioner's Index, The. Review of ...... ....................52, 196, 574
International System of Electro-Therapeutics, An. By Horatio R. Bigelow, M. D. Review of................ 289
Internationales Homöopathisches Jahrbuch. By Dr. Alexander Villers Review of......................... 488
Ironside, A S., M. D. Hypericum for Convulsions..................................... 568
It Do Move............................................ 568

Jackson, Frances, M. W., M. D. The Indicated Remedy in Diseases of Women.............................................. 372
Jackson's Obstetric and Gynæcological Pins. Notice of..................... 342
James, Bushrod W. Ideal Sanitation of a Physician's Office................ 548
James, Walter M., M. D. Aggravation from Jarring the Bed..................... 57
An Apology ........................................ 249

PAGE

James, Walter M., M. D. Apis-mellifica ... 105, 153, 201
Apology No. 2 ... 297
Arsenicum-album ... 345, 393, 441, 489, 537
In Memoriam. Dr. Mahlon Preston ... 501
Vaccination ... 1
Jarring of Bed, Aggravation from. W. M. James, M. D ... 57
Aggravation from. E. V. Ross, M. D ... 179
Jennings, Rev. C. P., S. T. D., M. D. In Memoriam. T. Dwight Stow, M. D ... 45
Johnson, G. W. The Acids of Fruits. 242
Jottings. C. Carleton Smith, M. D ... 176

Keaney, William, M. D. Morphine Habit Cured ... 195
Keith, Frederick S., M. D. Sycosis ... 204
Kennedy, A. L., M. D. Clinical Cases. 444
Kimball, Samuel A., M. D Capillary Bronchitis, with Brain Symptoms Cured by Stramonium ... 447
The Relative Value of Symptoms.. 250
Krause, Dr. C. H ... 198

Labor Pains Treated with the Simillimum. W. E. Everly, M. D ... 233
Ledyard, W. E., M. D. *The Organon* and Materia Medica Club of the Bay Cities of California ... 184, 517
Legal Medicine, Hamilton's Systems of. Notice of ... 53
Leonard, W. H., M. D. Evolution in Medicine, The Use of Association. 39
Leverson, Dr. M. R ... 391
Leverson, Montague B., M. D. Anti-Vaccination ... 433
Liberty of Science in Germany. B. Fincke, M. D ... 413
Lincoln, Dr. Guy A. T. In Memoriam ... 506, 565
Lippe, Mrs., Death of ... 575
Literary Digest, The. Review of ... 197
Lithæmia and Psora. Edward Cranch, M. D. ... 507

McClelland, President ... 53
MacLachlan, Dr. D. A. Has removed 536
MacLachlan & Brooks, Drs ... 535
McNeil, A. M., M. D. Cases Cured by Dr. Hesse ... 515
McNeil, A., M. D. Cases Cured by Alexander Villers, M. D ... 182
Primary, Indifferent, and Secondary Effects ... 299
Society of Homœopathicians ... 298
Where Homœopathy Failed ... 494
Manual of Genito-Urinary and Venereal Diseases, A. By Bukk G. Carleton, M. D. Review of ... 435, 566
Map of the World ... 248
Markham Sanatorium, The ... 152
Martin, Eleanor F., M. D. *The Organon* and Materia Medica Club of the Bay Cities of California ... 421
Materia Medica Notes ... 236
Materia Medica, Eclectic Syllabus of. 531
Materia Medica: Regional and Comparative. Review of ... 291
Materia Medica, Some Thoughts on. C. T. Schwenke, M. D ... 89
Materia Medica and Therapeutics. By John V. Shoemaker. Review of ... 570

Measles and Phenacetin: Which Killed the Patient—the Disease or the Treatment? Charles E. Page, M. D ... 94
Medical Examiner, The ... 295
Medicine, Practice of: Synopsis of. Review of ... 53
Menninger, C. F., M. D. Popular Fallacies ... 349
Mera, Dr. H. P. Has removed ... 535
Middletown State Homœopathic Hospital: Twenty-fourth Annual Report ... 532
Mind Symptoms of Our Homœopathic Drugs, The. J. R. Haynes, M. D. 450
Minnesota State Homœopathic Institute, The ... 200
Mitral Incompetency—With Consequent Pulmonary Lesion. S. Mills Fowler, M. D ... 467
Modern Gynæcology. By Charles H. Bushong, M. D. Review of ... 388
Moffat, John L., M. D. The Individual Cup at Communion ... 145
Morgan, A. R., M. D. International Hahnemannian Association. Address to Members ... 83
Morphine Habit Cured. Wm. Keaney, M. D ... 195
Morphine, Symptoms of. R. L. Thursston, M. D ... 563

National Medical College, The ... 248
National Society of Electro-Therapeutists ... 343
Natrum-muriaticum, Headache Cured by. J. T. O'Connor, M. D ... 567
Natrum sulphuricum, Aggravated Case of Neuralgia (Neuritis) of Fifth Nerve Relieved by. Joseph T. O'Connor, M. D ... 433
Nervous Debility. Sarah N. Smith, M. D ... 225
NewEngland Hahnemann Association, The ... 152
New York Homœopathic Medical Society. Transactions of ... 574
New York Homœopathic Union, The. Emma D. Wilcox ... 137
Norbury. Dr. Frank Parsons ... 440
Northern Indiana and Southern Michigan Society, The ... 239
Notes and Notices. 53, 151, 198, 248, 342, 391, 440, 535
Nursery Ethics. Mrs. Florence Hull Winterburn. Review of ... 390

Obstretical Surgery. By Egbert H. Grandin, M. D. Review of ... 146
O'Connor, Joseph T., M. D. Aggravated Case of Neuralgia (Neuritis) of Fifth Nerve Relieved by Natrum-sulphuricum ... 433
Headache Cured by Nat-mur ... 567
Olds, C. L., M. D. Two Acute Cases ... 98
*Organon* and Materia Medica Club of the Bay Cities of California. Eleanor F. Martin, M. D ... 421
*Organon* and Materia Medica Club of the Bay Cities of California, The. W. E. Ledyard, M. D ... 184, 421, 517, 552
Orificial Surgeons, American Association ... 391
Orificial Surgery ... 391
Our Senior Doctors. J. Bailey Sullivan, M. D ... 59

PAGE

Ovaries, Repertory of: Symptoms of. C. M. Boger, M. D. 540

Page, Charles E., M. D. Measles and Phenacetin: Which Killed the Patient—the Disease or the Treatment? 94
Paracelsus and Hahnemann. B. Fincke, M. D. 491
Patch, Frank W., M. D. Psora—Its Nature 108
Psora. Some Features of Its Treatment 405
Pathogenetic Materia Medica. Review of 437
Pease, F. O., Dr. Case reported in October number 561
Pease, F. O., M. D. Clinical Cases 458
Phillips, W. H., M. D. The Influence of Climate on Pulmonary Phthisis 512
Phthisis, The Influence of Climate on Pulmonary. W. H. Phillips, M.D. 512
Physicians' Insurance Association, The 294
Physician's Visiting List for 1896 573
Pierce, W. A. D., M. D. In Memoriam. Dr. Mahlon Preston 504
Plausible Fallacy, A. Edmund Carleton, M. D 401
Poke-berries, Notes on the Effects of, on Birds. W. E. Rotzell, M. D. 87
Popular Fallacies. C. F. Menninger, M. D. 349
Portrait of Bœnninghausen, A 294
Postscript 377
Potencies, High, The Value of. Theo. H. Winans, M. D 77
Practical Uranalysis and Urinary Diagnosis. By Charles W. Purdy, M. D. Review of 384, 571
Practice of Medicine, A Synopsis of the. By William B. Stewart, M. D. Notice of 53
Prescription Cards. By Stacy Jones, M. D. Review of 104
President McClelland 53
Preston, Frederic, M. D. Autopsy of Alexander III, Emperor of Russia 85
Preston, Mahlon, M. D. In Memoriam. Walter M. James, M. D 501
In Memoriam 504
Preston, Mahlon, M. D., Reminiscences of 569
Principles. Frank S. Davis, M. D 397
Protest from the I. H. A., A. J. H. Allen, M. D 36
Protest from the I. H. A., A. By L. B. Baylies, M. D 36
Psora, Lithæmia and 507
Psora, Its Nature. Frank W. Patch, M. D 108
Psora, Some Features of its Treatment, Frank W. Patch, M. D 405
Psychopathia Sexualis, Suggestive Therapeutics in. By Dr. A. Schrencknotzing 150, 245
Pulsatilla in Sleeplessness 434
Pure Homœopathy *vs.* Eclecticism. Joseph Fitz Mathew, M. D 43
Pyrosis, Graphites for 288

Quarterly Atlas of Dermatology. By H. Ohman Dumesnil, M. D. Review of 533

PAGE

Regional and Comparative Materia Medica. By John Gilmore, M. D. Review of 291
Regional and Comparative Materia Medica, The. Notice of 440
Relative Value of Symptoms, The. Samuel A. Kimball, M. D 250
Removal Notice. Note 53
Repertory of the Symptoms of the Ovaries. C. M. Boger, M. D 540
Repertory of Scarlet Fever. Edward Rushmore, M. D 60
Rhus Poisonings, Symptoms from. Robert A. Billings, M. D 369
Richardson, Wm. C., M. D. Homœopathy in Missouri 234
Richardson, Dr. Wm. C 200
Roast Pig 56
Rogers, Dr. L. D 199
Ross, E. V., M. D. Aggravation from Jarring 179
Rotzell, W. E., M. D. Notes on the Effects of Poke-berries on Birds. 87
Rules for the Proper use of Heraldry in the United States. By Bailey, Banks & Biddle. Review of 534
Rushmore, Edward, M. D. Repertory of Scarlet Fever 60

Safe from Ordinary Maladies 56
Salivary Indigestion 188
Sanitary Climatology. Mark W. Harrington 83, 285
Sarcocele, Aurum in 382
Satterlee, Dr. M. D., has removed 151
Scarlet Fever, Repertory of. Edward Rushmore, M. D 60
Scarlet Fever. Table of Abbreviations of Remdies used in the Repertory 72
Schneider, Dr. Nathaniel 200
Schwenke, C. T., M. D. Some Thoughts on Materia Medica 89
Science of Homœopathy, The. By Charles J. Hempel. Review of 386
Scientific American 574
Scientific American Reference Book. Review of 151
"Sennine" 199
Sepia 194
Sexual Neurasthenia, By George M. Beard, M. D. Review of 147
Shannon, S. F., M. D. The Eulogium of Dr. Dake 193
Silicea in Affections of the Eustacian Tube. Dr. U. Goullon 288
Smith, C. Carleton, M. D. Jottings 176
Smith, Sarah N., M. D. Nervous Debility 225
Two Cases of Sycosis 180
Society of Homœopathicians 300, 397, 444
Southern Homœopathic Medical Association 151, 392
Spasms and Convulsions. A. W. Holcombe, M. D 322, 378
Spitting of Blood 381
Standard Dictionary of Funk & Wagnalls 52, 242, 344, 390, 534
Standard School Books 390
Stanton, Lawrence M., M. D. Aortic Insufficiency 73
Cases from Practice 73
Cornu-cutaneum 75
Coryza 74
Flatulent Dyspepsia 75
Housemaid's Knee 76

PAGE

St. Louis Journal of Homœopathy. W. A. Edmonds, M. D., Ed. Review of ... 51
Stramonium. Capillary Bronchitis, with Brain Symptoms, Cured with. Samuel A. Kimball, M. D. 447
Stow, T. Dwight, M. D. Vaccination Denounced ... 525
Substitution: How We Intend to Check ... 576
Suggestive Therapeutics in Psychopathia Sexualis. By Dr. A. Schrencknotzing ... 150, 245
Sullivan, J. Bailey. M. D. Our Senior Doctors ... 59
Sycosis. Frederick S. Keith, M. D ... 204
Two Cases of. Sarah N. Smith, M. D ... 180
Syllabus of Eclectic Materia Medica and Therapeutics. Ed. by Harvey W. Felter, M. D. Review of ... 531
Symptoms from Morphine. R. L. Thurston. M. D ... 563
Syphilinum, or Luesinum, with Comments. J. H. Allen, M. D ... 353
System of Legal Medicine, A. By Allen McLane Hamilton, M. D. Review of ... 103

Tafel, the late A. J ... 200
Taft, Charles H., M. D. A Consideration of Some of the Objections Offered by Physicians to Amalgam Fillings ... 120
Taking the Case. J. A. Tomhagen, M. D ... 362
Tendon Grafting. S. E. Milliken, M. D ... 565
Therapetical Application of Peroxide of Hydrogen, Glycozone, and Hydrozone. Review by Charles Marchand ... 247
Therapeutic Hints. C. M. Boger, M. D ... 194, 241, 383
Therapeutics: Essentials of Homœopathic. By W. A. Dewey, M. D. Review of ... 572
Therapeutics: Materia Medica and: Review of ... 570
Thomson, J. W., M. D. Thoughts on the Philosophy of Homœopathy and Correlated Subjects ... 301
Thoughts on the Philosophy of Homœopathy and Correlated Subjects. J. W. Thomson, M. D ... 301
Tinospora-cordifolia ... 101
Tomhagen, J. A., M. D. Taking the Case ... 362
Tragical Historie of our Late Brother, Robert Earl of Essex, The. By Orville W. Owen, M. D. Review of ... 383
Transactions of the Antiseptic Club. Review of ... 390
Transactions of the Thirtieth Session of the Homœopathic Medical Society of the State of Pennsylvania. Review of ... 529
Truth about Homœopathy, The. By Dr, Wm. H. Holcombe ... 149
Truth Shall Make us Free, The. J. H. Allen, M. D ... 133, 263
Twenty-fourth Annual Report of the Middletown State Homœopathic Hospital. Review of ... 532

Unfortunate ... 199
Universal Homœopathic Annual of 1894. By Francois Cartier, M. D. Review of ... 527
University of Michigan, The ... 248
Uranalysis and Urinary Diagnosis, Practical. Review of ... 384
Urtica Moroides, or Laportea Moroides. Alfred Heath, M. D ... 100
Uterus, Interstitial Febroid of. Edmund Carleton, M. D ... 298

Vaccination ... 440
Vaccination. L. Hoopes, M. D ... 267
Vacciuation Denounced. T. Twight Stow, M. D ... 525
Vaccination, Dr. Constantine Hering on. W. B. Clark, M. D ... 232
Vaccination. Editorial. Walter M. James, M. D ... 1
Vaccination Law of the State of New York. Points on the Repeal of Compulsory. B. Fincke, M. D ... 228
Vaccination and Small-pox. Dr. D. N. Banerjee ... 321
Villers, Cases Cured by Dr ... 218
Vomiting of Fluids, but Not of Solids. A. H. Tompkins, M. D ... 563

Wilcox, Emma D. The New York Homœopathic Union ... 137
Winans, Theo. H., M. D. The Value of High Potencies ... 77
Woman's International Provers' Association, The ... 392
Women, The Indicated Remedy in Diseases of. Frances M. W. Jackson, M. D ... 372
Wrong Diagnosis ... 56

Yingling, W. A., M. D. Repertory of Appendicitis ... 273
Yingling: Accoucher's Emergency Manual ... 196

VOL. XV. JANUARY, 1895. No. 1.

### THE HOMŒOPATHIC PHYSICIAN,

A Monthly Journal of Homœopathic Materia Medica and Clinical Medicine.

*"He is a freeman whom truth makes free, and all are slaves beside."*

*"Seek the Truth: come whence it may, cost what it will."*

EDITED AND PUBLISHED BY

WALTER M. JAMES, M. D.

NOTICE.

*All articles for publication, books for review, business communications, etc., must be sent to Editor, 1231 Locust Street, Philadelphia.*

*Subscribers will please notice that this Journal will be sent until arrears are paid and it is ordered discontinued.*

PHILADELPHIA:

